# Ragopathies and the rising influence of RagGTPases on human diseases

**DOI:** 10.1038/s41467-024-50034-4

**Published:** 2024-07-10

**Authors:** Irene Sambri, Marco Ferniani, Andrea Ballabio

**Affiliations:** 1 Telethon Institute of Genetics and Medicine (TIGEM), Pozzuoli, (NA), Italy; 2 Scuola Superiore Meridionale (SSM, School of Advanced Studies), Genomics and Experimental Medicine Program (GEM), Naples, Italy; 3 Department of Molecular and Human Genetics, Baylor College of Medicine, Houston, TX USA; 4 Jan and Dan Duncan Neurological Research Institute, Texas Children’s Hospital, Houston, TX USA; 5 Medical Genetics Unit, Department of Medical and Translational Science, Federico II University, Naples, Italy

**Keywords:** Mechanisms of disease, Preclinical research, Gene regulation

## Abstract

RagGTPases (Rags) play an essential role in the regulation of cell metabolism by controlling the activities of both mechanistic target of rapamycin complex 1 (mTORC1) and Transcription factor EB (TFEB). Several diseases, herein named ragopathies, are associated to Rags dysfunction. These diseases may be caused by mutations either in genes encoding the Rags, or in their upstream regulators. The resulting phenotypes may encompass a variety of clinical features such as cataract, kidney tubulopathy, dilated cardiomyopathy and several types of cancer. In this review, we focus on the key clinical, molecular and physio-pathological features of ragopathies, aiming to shed light on their underlying mechanisms.

## Abstract (web summary)

RagGTPases (Rags) play an essential role in the regulation of cell metabolism by controlling the activities of both mechanistic target of rapamycin complex 1 (mTORC1) and Transcription factor EB (TFEB). Here the authors review several diseases, termed ragopathies, that are associated with Rag dysfunction.

## Current knowledge on Rags function

Heterodimeric Ras-related small GTP-binding proteins (Rag-GTPases) comprise four related small guanosine triphosphatase (GTPase) proteins that bind mTORC1 in an amino acid-dependent manner and serve as crucial regulators of its kinase activity towards a variety of substrates^1,2^. mTORC1 has a dual role in the regulation of cell metabolism: it promotes cell anabolism by activating S6 kinase (S6K) and by inhibiting the eukaryotic initiation factor 4E-binding protein 1 (4E-BP1)^3–6^, and it hinders cell catabolism by inhibiting the Unc-51-like kinase 1 (ULK1)^7,8^ and by blocking the nuclear translocation of Transcription Factors EB and E3 (TFEB and TFE3)^9–13^, which are members of the MiT-TFE family and master transcriptional regulators of lysosomal biogenesis and autophagy^9–13^.

Four Rag proteins (RagA, RagB, RagC, RagD) have been identified in mammals. RagA and B are orthologous to yeast Gtr1, while RagC and D to yeast Gtr2^14–19^. The Rags perform their function as heterodimers, which may be composed by either RagA or B that interact with either RagC or D, thus resulting in four types of heterodimers: RagA/RagC, RagA/RagD, RagB/RagC, and RagB/RagD. All Rags feature an N-terminal canonical GTPase domain that consists of two switches (I and II), which are necessary for magnesium (Mg^+^) coordination and nucleotide binding^20–22^, and a C-terminal domain composed of 5 β-sheets flanked by 2 α-helical layers, which mediates Rag dimerization b^18–20^. Rags localize to the cytosolic side of the lysosomal surface by binding to the pentameric ragulator complex composed of LAMTOR1 (also known as p18), LAMTOR2 (p14), LAMTOR3 (MP1), LAMPTOR4 (C1ORF59), and LAMTOR5 (HBXIP)^20,22,23^.

The function of Rags is regulated by amino acid levels in the cells. When intracellular amino acid concentration is high, RagA and B are loaded with GTP, while RagC and D with GDP. This leads to the formation of four types of dimer configurations (i.e. RagA or B-GTP/RagC or D-GDP). These configurations facilitate the interaction between RagA or B and RAPTOR, promoting lysosomal recruitment of mTORC1^19,21,24^, which allows its interaction with Rheb, a GTP-binding protein that promotes mTORC1 kinase activity site towards S6K and 4E-BP1^25,26^. Conversely, in low-nutrient conditions, RagA and B are loaded with GDP, while RagC and D with GTP. This causes a conformational change that promotes the dissociation of mTORC1 from the lysosomal membrane^19,21,24^. While the GTP loading of RagA and B is essential for lysosomal recruitment of mTORC1, the GDP loading of RagC and D plays a lesser role in this process. Recent studies have shown that the GDP loading RagC and D is crucial to promote mTORC1-mediated phosphorylation of TFEB and other MiT-TFE factors^27,28^. Thus, only RagA(B)-GTP/RagC(D)-GDP configurations can promote the phosphorylation of MiT-TFE factors, whereas both RagA(B)-GTP/RagC(D)-GDP and RagA(B)-GTP/RagC(D)-GTP configurations can promote the phosphorylation of S6K and 4E-BP1^29^. These findings indicate that the Rags, which have long been known to bind to MiT-TFE factors^30^, enable substrate specificity of mTORC1 activity^28^. The mechanism underlining such substrate specificity relies on the ability of GDP-loaded RagC and D to perform substrate recruitment for mTORC1 by binding directly to MiT-TFE factors, but not to S6K and 4E-BP1^28^. The recent determination of the cryo-EM structure of the Ragulator-Rags-mTORC1-TFEB megacomplex has provided structural evidence that TFEB is recruited and phosphorylated by mTORC1 in a RagC-GDP-dependent manner^31^.

As with other small G proteins, the nucleotide binding status of the Rags is regulated by GTPase-activating proteins (GAP), which accelerate the conversion of GTP to GDP. RagA and B nucleotide binding is regulated by the GATOR1 complex, which consists of three subunits: nitrogen permease regulator-like 2 (NPRL2), nitrogen permease regulator-like 3 (NPRL3), and DEP domain-containing 5 (DEPDC5)^32^. GATOR1 functions as a negative regulator of mTORC1 activity by promoting the RagA-GDP state^32,33^. The genetic ablation of any GATOR1 subunit leads to permanent lysosomal localization of mTORC1, even in amino acid-depleted conditions. This results in constitutive (i.e. amino acid-independent) mTORC1 activity^32,33^. In mammals, the activity of GATOR1 requires the KICSTOR protein complex as co-factor, which is composed of the KPTN, ITFG2, C12ORF66, and SZT2 subunits, and acts as a scaffold to recruit GATOR1 to the lysosomal surface^34^. The GAP activity of GATOR1 is finely regulated by other protein complexes, such as GATOR2, CASTOR, and SAMTOR. The v-ATPase also plays a role in the regulation of Rag A and B via an incompletely understood mechanism^7,32,33,35–42^.

RagC and D GDP binding is dependent on FLCN, which acts as a GAP that converts RagC/D-GTP into RagC/D-GDP^43–46^. FLCN activity requires the formation of a protein complex, composed by FLCN and FLCN-interacting protein 1 (FNIP1) or FNIP2. During amino acid starvation, the FLCN complex is recruited to the lysosome and interacts with inactive RagA or B (RagA(B)-GDP), forming a specific configuration named the Lysosomal Folliculin Complex (LFC)^44,45,47,48^. This represents an inactive configuration because the catalytic domain of FLCN is kept away from RagC(D) nucleotide-binding pockets^44,45^. Once amino acids are replenished, inhibition of GATOR1 leads to GTP binding of RagA, disrupting the LFC configuration and promoting the formation of the Active Folliculin Complex (AFC). This allows positioning of FLCN catalytic site close to RagC(D) nucleotide pocket^46^.

**Fig. 1:**
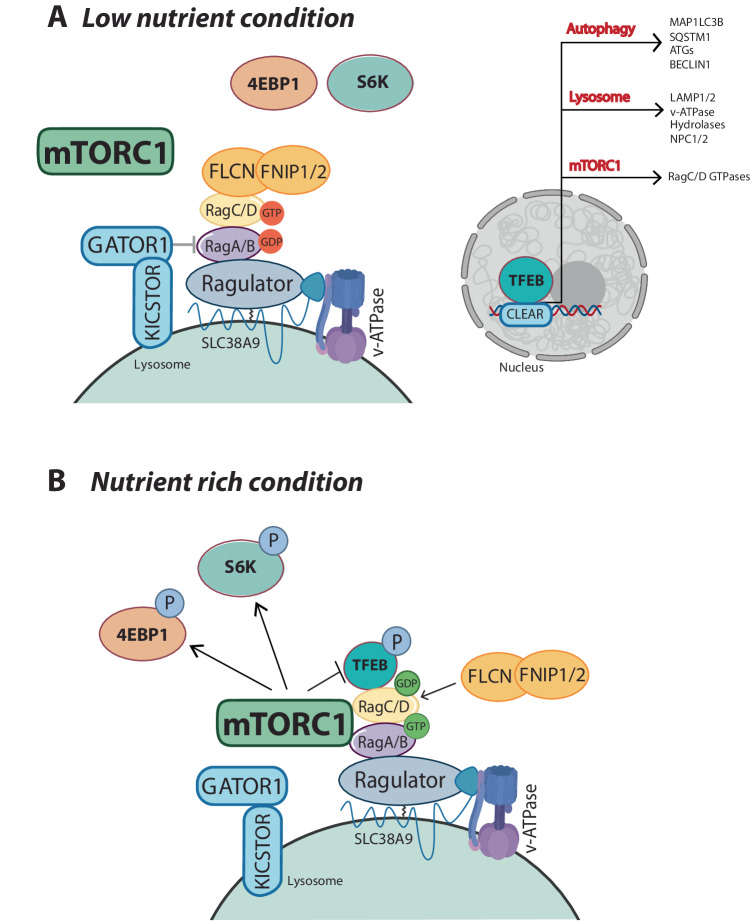
Regulation of Rag GTPase Activity in the Presence or Absence of Amino Acids. **A** *Low nutrient condition*: During nutrient deprivation, GATOR1, with its co-factor KICSTOR, promotes the GDP-loading of RagA/B, preventing the lysosomal recruitment of mTORC1. Conversely, nutrient deprivation promotes lysosomal recruitment of FLCN, which inhibits its GAP activity toward RagC/D. The resulting RagA/B-GDP;RagC/D-GTP heterodimers lead to mTORC1 inactivation and promote TFEB nuclear translocation. TFEB binds to CLEAR sequences found within the promoter of its target genes, exerting control over multiple pathways and cellular functions such as autophagy, lysosomal biogenesis, and mTORC1 signaling, the latter being controlled through the upregulation of RagC/D GTPases. **B** *Nutrient rich condition*: Nutrients in the lysosomal lumen inhibit GATOR1 and promote mTORC1 lysosomal recruitment through Rag-dependent mechanisms mediated by interaction between RagA/B with Raptor. This condition allows the activity of mTORC1 towards S6K and 4E-BP1 and activates the GAP activity of FLCN toward RagC/D, favoring their GDP-loading. The resulting RagA/B GTP–RagC/D-GDP heterodimers enable the recruitment of TFEB to mTORC1.Figure created with BioRender.com, released under a Creative Commons Attribution-NonCommercial-NoDerivs 4.0 International license.

The function of FLCN is crucial for mTORC1-mediated phosphorylation of TFEB and other MiT-TFE factors but is dispensable for the phosphorylation of S6K and 4E-BP1. This is consistent with the evidence that RagC(D) GDP loading is specifically required for TFEB and TFE3 recruitment by mTORC1^27,28,45,49^. Interestingly, while Rheb plays a crucial role for the phosphorylation of S6K and 4E-BP1 it appears to be dispensable for the phosphorylation of TFEB and TFE3^28^. These findings indicate the presence of two distinct branches in the regulation of mTORC1 signaling: a “canonical” one that requires Rheb and is involved in the phosphorylation of S6K and 4EBP1, and a “non-canonical” branch that requires FLCN and is involved in the phosphorylation of MiT-TFE factors^29^. Figure 1 shows the main regulators of Rag activity under nutrient-rich and low-nutrient conditions.

## Ragopathies caused by mutations of genes encoding RagGTPases

Consistent with their prominent role in modulating cell metabolism, an imbalance in the function of Rags results in an alteration of mTORC1 signaling, leading to group of heterogeneous pathological conditions herein defined as “ragopathies”. Dysfunction of Rags could be the result of mutations of either genes encoding the Rags, or of genes encoding their upstream regulators. Interestingly, all described disease-causing mutations that occur in genes encoding the Rags appear to be gain-of function leading to enhanced activity of the Rags. In most cases, affected individuals are germ-line heterozygous for the disease-causing mutations and the diseases are inherited in an autosomal dominant manner. One prominent exception is the case of follicular lymphoma in which *RRAGC* gene mutations occur somatically and, therefore, the disease is not inherited.

### RRAGA mutations in cataract

**Fig. 2:**
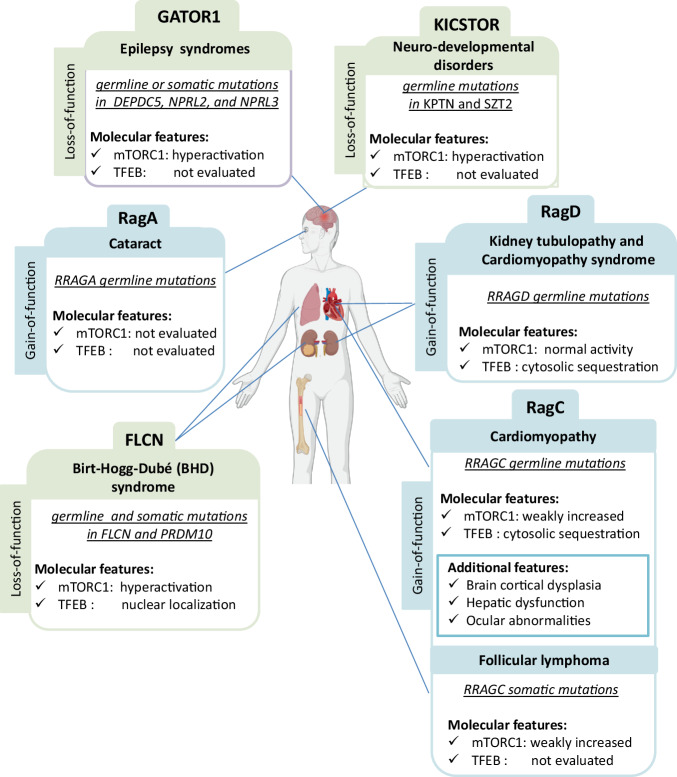
Diseases arising from genetic mutations in Rag GTPases and their regulators. The figure provides a schematic representation of the organs primarily affected in conditions resulting from human mutations in the Rags and their regulators, highlighting the impact of their mutations on mTORC1 and TFEB activity. Gain of Function mutations in Rag GTPases: Germinal mutations in *RRAGA* are associated with cataracts, but the impact on mTORC1 and TFEB activity remains unexplored. Somatic mutations in *RRAGC*, which are linked to follicular lymphoma, lead to a modest increase in mTORC1 activity. Germline mutations in *RRAGC*, causing cardiomyopathies, (with possible additional features as brain cortical dysplasia, hepatic dysfunction or ocular abnormalities), result in the cytosolic sequestration of TFEB. Nevertheless, the effect on mTORC1 activity toward its canonical substrates appears to be only partially increased. Germline mutations in *RRAGD* do not affect canonical mTORC1 signaling but lead to cytosolic sequestration of TFEB. Loss of function mutations in Rag GTPases regulators: Germline or somatic mutations in GATOR1 subunits (*DEPDC5, NPRL2/3*) associated with epilepsy syndrome result in an upregulation of canonical mTORC1 signaling. Germline mutations in KICSTOR subunits (*KPTN* and *SZT2*), leading to neuro-developmental disorders, also cause an upregulation of canonical mTORC1 signaling. Germline and somatic mutations in Folliculin (*FLCN*) or PRDM10 are causative of Birt-Hogg-Dubé syndrome. These mutations result in an upregulation of canonical mTORC1 signaling and lead to a constitutively nuclear localization of TFEB. Figure created with BioRender.com, released under a Creative Commons Attribution-NonCommercial-NoDerivs 4.0 International license.

The first demonstration of a germline mutation in a Rag causing an inherited human condition was the identification of a *RRAGA* mutation in a family with autosomal dominant cataract (Fig. 2)^50^. Whole Exome Sequencing (WES) revealed a heterozygous missense mutation that co-segregated with progressive juvenile-onset posterior sub-capsular cataracts. This mutation affects the Leu60 residue, which is located near the Mg^2+^ binding site, resulting in enhanced ability of RagA to bind GTP and activate mTORC1. The same gain-of-function mutation was also identified in an unrelated patient with juvenile-onset cataract. Subsequently, another mutation in the RRAGA 5’-UTR (c.−16G > A) was found in a third unrelated patient with congenital cataract. Functional studies in human lens epithelial cells showed that the *RRAGA* Leu60Arg mutant co-localized with lysosomes more efficiently compared with the *wild type* form. The authors also observed a reduced downstream LC3B-II/LC3B-I ratio, suggesting a potential downregulation of autophagy by an unclarified mechanism^50^. Unfortunately, the impact of the RagA Leu60Arg mutation on the overall function of mTORC1 was not explored. However, the suggestion that *RRAGA* mutations lead to autosomal dominant cataracts by disrupting autophagy is in line with the prominent role that autophagy plays in lens development and cataract formation^51,52^. Currently, there are no animal models specifically tailored to study of RagA mutations in the eye. However, the generation of conditional RagA KO mice, RagB KO mice, and RagA/B double KO mice has provided valuable insights into the intricate interplay and compensatory mechanisms between RagA and RagB across different tissues and developmental stages. The phenotype and main molecular features of Rags animal models are listed in Tables 1–3.

**Table 1:** RagA GTPase animal models and relative phenotype

	*Genotype*	*Phenotype*	*References*
*Mouse*	RagA CMV-cre (KO)	Embryo lethality (E10.5), developmental abnormalities (open neural tube and reduced size), reduction of mTORC1 activity.	*Efeyan A*, et al.^110^
	RagA CMV-cre (iKO) tamoxifen-inducible	Mice succumbed within 3 weeks, myeloid cell expansion (monocyte leukemia).	*Efeyan A*, et al.^110^
	RagA Alb-cre	Downregulation of mTORC1 signaling.	*Efeyan A*, et al.^110^
	RagA^Q66L^ or RagA^GTP/GTP^(KI) C57BL/6:129 Sv	Normal development, 100% neonatal death.	*Efeyan A*, et al.^64^
	RagA^Q66L^ orRagA^GTP/GTP^ (KI) C57BL	Developmental defects (cranial abnormalities, hypopigmentation), 100% neonatal death.	*de la Calle Arregui C*, et al.^63^
	RagA^Q66L^ orRagA^GTP/Δ^ (KI) C57BL/6:129 Sv	Normal development, 60% neonatal survival, complete insensitivity to nutrient withdrawal, impaired B cell function.	*Efeyan A*, et al.^110^
	RagA^Q66L^ orRagA^GTP/GTP^C57BL6/J, B1-8i	Impaired B cell activation.	*Ersching J*, et al.^111^
*Zebrafish*	Rraga^st77/st77^ (KO)	Reduced microglia with expanded lysosomal compartment but inability to digest apoptotic neuronal debris.	*Shen K*, et al.^112^
*Drosophila*	RagA^TN (dominant negative)^	Diminished synaptic growth (decreased bouton numbers).	*Wong CO*, et al.^113^

**Table 2:** RagA/B and RagB GTPases animal models and relative phenotype

	*Genotype*	*Phenotype*	*References*
*Mouse*	RagA /RagB CMV-cre (DKO)	Embryo lethality (E10.5), complete inactivation of mTORC1 nutrient signaling.	*Efeyan A*, et al.^110^
	RagA/RagB Alb-cre	Complete inactivation of mTORC1 nutrient signaling.	*Efeyan A*, et al.^110^
	RagA/RagB Mck-cre	No phenotype in skeletal muscle but cardiac hypertrophy, increase of mTORC1 signaling in cardiomyocite and defects in autophagic flux.	*Kim YC*, et al.^67^
	RagB CMV-cre (KO)	Normal mendelian ratio, mTORC1 activity unaffected.	*Efeyan A*, et al.^110^

**Table 3:** RagC GTPase animal models and relative phenotype

	*Genotype*	Phenotype	*References*
*Mouse*	RagC ^Q119L/+^ hypomorphic	Attenuated mTORC1 nutrient signaling, impaired B cell function.	*Ortega-Molina A*, et al.^114^
	RagC ^T90N/+^, RagC ^S75C/+^ (KI)	Partial insensitivity to nutrient deprivation, overactive B cell function.	*Ortega-Molina A*, et al.^54^
*Zebrafish*	RagC^S56Y^ (KI)	Cardiac hypertrophy, TFEB cytosolic sequestration, altered autophagy.	*Kim M, Lu L*, et al.^58^
*Drosophila*	RagC^Δ^ (KO)	Diminished synaptic growth (fewer boutons in neuromuscular junctions (NMJ)).	*Shen K*, et al.^112^

### RRAGC mutations in dilated cardiomyopathy

Long et al. reported the first case of a de novo missense germline heterozygous mutation (S75Y) in the *RRAGC* gene, which was identified by whole-exome sequencing in a fetus with a syndromic dilated cardiomyopathy^53^. The expression of mutant *RRAGC* in AD293 cells resulted in enhanced S6K phosphorylation during fasting compared with *wildtype* RagC suggesting a dysregulation of the mTORC1 pathway. More recently, an extensive study based on genomic sequencing in children with dilated cardiomyopathy identified several germline mutations in the *RRAGC* gene affecting amino acids Ser75, Thr90, Tyr115, and Pro118, which are located within the nucleotide-binding domain. Interestingly, the same amino acids were also found to be mutated somatically in follicular lymphoma (see below) (Fig. 2)^54–57^. Clinical manifestations of patients carrying germline *RRAGC* mutations were not limited to the heart and included brain cortical dysplasia, hepatic dysfunction and ocular abnormalities, such as cataract and microspherophakia. Metabolic abnormalities, including lactic acidosis and hyperammonemia, potentially arising from a combination of secondary mitochondrial dysfunction and organ failure, were also described. Studies performed in fibroblasts from a patient carrying a RagC Thr90Asn mutation revealed a TFEB cytosolic localization both in fed and starved cells and a slight increase of mTORC1 activity as measured by analyzing S6K phosphorylation^55^. A zebrafish model carrying an S56Y mutation in the *Rragc* gene, which resembles the human S75Y mutation linked to dilated cardiomyopathy, was generated using TALEN gene editing^58^. At 7 months of age this zebrafish model exhibited decreased ventricular systolic function, enlarged ventricular surface area, and reduced density of ventricular trabeculae. Mutant hearts showed downregulation of lysosomal genes, which are known to be TFEB targets. Notably, ectopic expression of TFEB improved survival of this fish model, whereas depleting *mTOR* had no effect^58^. These observations align with the concept that the S75Y gain-of-function mutation in the *Rragc* gene specifically enhances the phosphorylation of TFEB by mTORC1, thus promoting its cytosolic sequestration, whereas it has no effect on mTORC1 activity on other substrates^59^.

### Somatic RRAGC mutations in follicular lymphoma

Follicular lymphoma is the second most common form of non-Hodgkin lymphomas (NHL)^60^. The genetic hallmark of follicular lymphoma is the t(*14*;*18*)(*q32*;*q21*) chromosomal aberration, which leads to the overexpression of the anti-apoptotic protein Bcl2 and is found in more than 85% of all follicular lymphoma cases^60^. However, other oncogenic events in addition to Bcl2 overexpression are required to promote FL development and are known affect disease progression. Somatic mutations in *RRAGC* have been identified in 9 to 17% of patients with follicular lymphoma^56,57^. These mutations were not detected in various hematological malignancies, including myeloid disorders and other mature B cell non-Hodgkin lymphoma entities, suggesting that they are likely to be pathogenic specifically for follicular lymphoma (Fig. 2). Most of these mutations are missense resulting in gain-of-function and are concentrated within the nucleotide-binding domain, with key hotspots at the evolutionarily conserved amino acids Ser75, Thr90, Tyr115, Asp116, Pro118, Gly119. Experiments performed in transfected HEK293T cells^57^ and in stable lentiviral-transduced B-cell lymphoma cell lines overexpressing RagC mutants^56^ showed a slight increase of S6K phosphorylation during starvation. However, given the high variability of the results, this aspect needs to be further investigated. Notably, one of these mutations, Ser75Y, highly resembled the S75L mutation previously shown to enhance RagC-TFEB interaction and to promote TFEB phosphorylation and cytoplasmic localization^28^. However, due to the established evidence that in several other malignancies, such as renal cancer, the MiT/TFE factors act as oncogenes^61^, whether and how RagC activation promotes lymphomagenesis via TFEB regulation remain to be clarified. More than 50% of patients carrying *RRAGC* mutations also carried mutations in ATP6V1B2 and ATP6AP^56^, whose genes encode components of the vacuolar H^+/-^ATP ATPase (V-ATPase), an enzyme crucial for the amino acid-induced activation of mTORC1^62^. Considering the functional connection of the V-ATPase complex with Rag GTPases and Ragulator in sensing amino acids and activating mTORC1 signaling^62^, the simultaneous occurrence of *RRAGC* mutations alongside mutations in either ATP6V1B2 or ATP6AP1 raises the question of whether there is functional interplay. However, experimental clarification is still needed to validate this hypothesis.

Knock-in models of RagC mutations Ragc^T89N^ and Ragc^S74C^ (corresponding to human T90N and S75C, frequently observed in human follicular lymphoma), were generated to explore the mutations’ oncogenic potential and their impact on B cell responses^54^. Mice carrying RagC^S74C/+^ and RagC^T89N/+^ mutations were obtained in sub-Mendelian ratios, indicating that partially penetrant lethality occurs before weaning. Additionally, breeding heterozygous RagC^S74C/+^ or RagC^T89N/+^ mice did not result in viable homozygous neonates. These findings were not surprising, considering that mice expressing a homozygous constitutively active form of RagA (Rag^AQ66L^) exhibited fully penetrant neonatal lethality^63,64^. Heterozygous RagC mutant mice did not display evident phenotypic alterations^54^, whereas crossing RagC mutant S74C and T89N strains with VavP-Bcl2 transgenic strains (a standard genetic tool to generate follicular lymphoma in mice) accelerated the development of follicular lymphoma^54^. These results coupled with the findings that *RRAGC* mutations are involved in the development of human follicular lymphoma and underscore the significance of Rag signaling in lymphomagenesis. However, the mechanism by which RagC promotes lymphomagenesis remains unclear.

### RRAGD mutations in kidney tubulopathy and dilated cardiomyopathy

Schlingmann at al. reported several families in which affected individuals showed kidney tubulopathy and dilated cardiomyopathy and carried heterozygous *RRAGD* missense mutations at conserved residues in GTP-binding domains (S76L, T97P, P119L, I221K). This was the first study to underscore the role of RagD in renal electrolyte handling and cardiac function^65^. A more recent study, reported a new family with a similar phenotype carrying a novel mutation (P88L) in the *RRAGD* gene and demonstrated that all reported *RRAGD* disease-causing mutations induce auto-activation of RagD (Fig. 2), which occurred independently of folliculin (FLCN), the GTPase-activating protein responsible for RagC/D activation^59^. Expression of these auto-activating RagD mutants in cultured cells resulted in the constitutive phosphorylation of TFEB and TFE3 by mTORC1, which inhibited TFEB and TFE3 nuclear translocation and transcriptional activity, with little or no effect on the activity of mTORC1 towards other substrates such as S6K and 4E-BP1. Similar results were obtained in patient-derived RagD P88L fibroblasts^59^, indicating that disease-causing RagD mutations specifically affect TFEB and TFE3, the non-canonical branch of the mTORC1 pathway^29^. These results align with previous studies highlighting the relevance of RagC/D activity in the regulation of mTORC1 activity towards TFEB and TFE3 and a marginal role in the regulation other substrates^28,29,46,49,66^. Therefore, it appears that in kidney tubulopathy and cardiomyopathy the inhibition of TFEB and TFE3, rather than hyperactivation of mTORC1, is the key driver of the disease phenotype^59^. These findings are consistent with previously described studies that used a zebrafish model of cardiomyopathy carrying the RagC S56Y mutation, in which ectopic expression of TFEB improved survival^67^. Unfortunately, there are no established mouse models specifically designed to study the function of RagD in physiological and pathological conditions. This underscores the importance of future research, especially considering the steadily increasing identification of mutations in *RRAGC* and *RRAGD* in human diseases.

## Ragopathies caused by mutations in regulators of the Rags

As discussed above, the activity of the Rags is finely regulated to allow the Rags to respond to a variety of stimuli, such as nutrient availability, cellular stress, and metabolic needs of specific cell types. Indeed, Rag activity is subject to the control exerted by several proteins and protein complexes. Both germline and somatic disease-causing mutations have been identified in negative regulators of the Rags. While the disease-causing mutations that were identified in the Rags lead to gain-of-function, those identified in regulators of the Rags are associated with loss-of-function.

### GATOR1 mutations in focal epilepsy

RagA and B function is controlled by the GAP activity of the GATOR1 protein complex, which is composed of three subunits: DEPDC5, NPRL2, and NPRL3. Deletion of any of these three subunits leads to the constitutive activation of mTORC1 even in amino acid-depleted conditions^32^. Loss-of-function mutations in each of the three GATOR1 subunits have been identified in patients with several types of epilepsy and seizure-associated disorders, consistent with the important role of mTORC1 in neuronal function and plasticity (Fig. 2)^68–73^. DEPDC5-related epilepsy is an autosomal dominant disorder with affected individuals carrying germline heterozygous mutations. Some patients have biallelic variants in DEPDC5, and some are mosaic for a somatic DEPDC5 variant that occurred in the brain. Generally, individuals with a heterozygous nonsense or frameshift pathogenic variant leading to a premature stop codon are more likely to have brain malformations (focal cortical dysplasia or hemimegaloencephaly). Individuals with germline biallelic pathogenic variants and a severe multisystemic phenotype are very rare, with only a few individuals having been reported. Asymptomatic heterozygotes are common in families with DEPDC5-related epilepsy and penetrance could be as low as 60%^74,75^. Consequently, DEPDC5-related epilepsy encompasses a range of epilepsy syndromes, almost all of which being characterized by focal seizures, with seizure onset in a discrete area of the brain. While about 60% of individuals with DEPDC5-related epilepsy have a normal brain magnetic resonance imaging (MRI)^76^, some have cortical malformations, such as focal cortical dysplasia type II or hemimegalencephaly^74,75^.

Most of the mutations in the DEPDC5 gene result in the production of truncated proteins that undergo rapid degradation, thus altering the formation of the GATOR1 complex, ultimately leading to mTORC1 hyperactivity. The mechanism by which mTORC1 hyperactivity promotes the occurrence of focal seizures is unclear. It has been suggested that mTORC1 hyperactivity alters neuronal synaptic activity, thus triggering seizures. Homozygous *Depdc5 KO* mice show embryonic lethality, while heterozygotes are viable and do not manifest increased susceptibility to seizures^73,77^. Conditional, neuron-specific, *Depdc5* KO mice (*Depdc5* fl/fl Syn-Cre) survive to adulthood and exhibit a progressive neurological phenotype, with larger brain, increased cortical neuronal size and astrogliosis^71^. Neurons collected from these mice showed constitutive activation of mTORC1 signaling pathway. Similarly, Depdc5 deletion in the dorsal telencephalon (Emx1-Cre, *Depdc5* fl/fl) showed spontaneous seizures and enlarged neurons with hyperactivation of mTORC1 signaling^78^. Interestingly, Ribierre and colleagues generated brain-specific mosaic Depdc5 *knock-out* (Depdc5fKO) mice, which showed abnormalities of neuronal migration to the cortical plate at E18.5, which was partially rescued by rapamycin treatment. Consistently, Depdc5 KO neurons showed high mTORC1 activity^71,73,79^. Loss of function mutations in the NPRL2 and NPRL3 genes have also been detected in individuals with autosomal dominant focal epilepsy^68,72,80–83^. Germline Nprl2 and Nprl3 KO mice are embryonic lethal, as observed for *Depdc5* KO mouse models^84^ and deletion of either Nprl2 or Nprl3 in the dorsal telencephalon using Emx1-Cre results in a similar phenotype to the one observed in Emx1-Cre *Depdc5* fl/fl mice^84^.

Disease-causing mutations have also been described in genes encoding subunits of the KICSTOR complex, which cooperates with the GATOR1 complex in the regulation of Rag A and B activity. To date, mutations have been identified in two KICSTOR subunits: KPTN and SZT2 (Fig. 2). KPTN mutations were initially reported in an Amish family with an autosomal recessive neurodevelopmental disorder characterized by macrocephaly and global developmental delay associated with seizures^85,86^. Likewise, biallelic variants in SZT2 also induce an autosomal recessive neurodevelopmental disorder characterized by severe epileptic encephalopathies and macrocephaly^87,88^. Both *Kptn* and *Szt2* KO mice accurately recapitulate the phenotype of the corresponding human diseases. They display cognitive and behavioral impairments, brain overgrowth, and hyperactive mTORC1 signaling in the brain^89,90^.

### Mutations in FLCN and PRDM10 in Birt–Hogg–Dube’ syndrome

As described above, FLCN is a GAP specific for RagC and D, which is essential for their activities. Loss-of-function mutations in the *FLCN* gene cause Birt-Hogg-Dubè (BHD) syndrome^91–93^, a rare inherited cancer syndrome that is transmitted as an autosomal dominant trait and is characterized by fibrofolliculomas, renal cysts, tumors and pneumothorax (Fig. 2)^94–96^. Kidney tumors in BHD syndrome are associated with loss of heterozygosity (LOH) due to somatic second hit mutations of the *FLCN* gene. BHD-like syndrome has also been reported, as part of a much more complex phenotype, in patients with Smith-Magenis syndrome, which carry large deletions of chromosome 17 including the FLCN gene^97–99^. Recent studies showed that BHD syndrome can also be caused by loss-of-function mutations of the *PRDM10* gene, which encodes an essential transcriptional regulator of FLCN expression^100,101^. Similarly, to other inherited cancer conditions, BHD syndrome is characterized by mTORC1 hyperactivation. This was thought to be a paradox, considering the previously described role of FLCN as positive regulator of mTORC1^28,43,47,48,66,102^. This paradox was recently solved thanks to the efforts of several groups who showed that FLCN activity is not required for mTORC1-mediated phosphorylation of “canonical” mTORC1 substrates, whereas it is essential for the phosphorylation of TFEB and TFE3, members of the MiT-TFE family of transcription factors^28,45,46,66^. Indeed, FLCN mutations in BHD syndrome impair RagC and D-mediated recruitment of TFEB and TFE3 by mTORC1, leading to their constitutively nuclear localization^28^. In turn, constitutively active TFEB and TFE3 cause mTORC1 hyperactivation through a previously described mTORC1-TFEB feedback loop^103^.

Recent studies based on the use of both transgenic mice and mouse xenografts demonstrated that constitutive activation of TFEB and TFE3 plays a crucial role in driving kidney cystogenesis and tumorigenesis associated with BHD syndrome^61^. *Tfeb* deletion in kidney-specific *Flcn* KO mice completely prevented kidney cyst formation, renal dysfunction and early mortality and also rescued mTORC1 hyperactivation in kidney tissue^28^. Most importantly, silencing of either TFEB or TFE3 abolished the ability of the UOK257 cell line, derived from a renal cancer of a patient with BHD syndrome^104^, to generate subcutaneous tumors in cell line-derived mouse xenografts^61^. These data raised the possibility that TFEB and TFE3 may drive kidney cancer in other inherited conditions. Tuberous Sclerosis Complex (TSC), an inherited cancer syndrome caused by mutations in either the TSC1 or TSC2 genes shares several similarities with BHD syndrome, such as the multisystemic phenotype, the presence of renal cysts and cancer and the association with mTORC1 hyperactivation^105,106^. Similarly, to *Flcn* KO cells, TSC2 KO cells also showed a nuclear localization of TFEB and TFE3 and an induction of their target genes associated with an expansion of the lysosomal system^107,108^. Remarkably, deletion of *Tfeb* in kidney-specific TSC2 KO mice prevented kidney cyst and tumor formation as well as early lethality and led to normalization of mTORC1 activity levels^109^. Together these studies clearly indicate the relevance of TFEB and TFE3 in renal cystogenesis and tumorigenesis.

## Concluding remarks and future directions

The adaptation of cell metabolism to environmental cues plays a crucial role in a variety of processes such as cell growth, proliferation, differentiation, and survival. In the past few years, Rag proteins have emerged as key regulators of cell metabolism due to their role in sensing environmental cues, decoding cellular metabolic needs, and mediating an adaptative metabolic response. The main direct “effector” of the regulatory function of Rags is mTORC1, which plays a pivotal role in promoting cell anabolism and growth and inhibiting cell catabolism. It is well established that the Rags regulate mTORC1 activity by mediating its recruitment to the surface of lysosomes^2,21,22^. Thus, lysosomes, long recognized as the pivotal mediators of cell catabolism, are now seen as hubs where the main players involved in intracellular nutrient sensing exert their function. Recent studies have introduced another level of complexity to our understanding of how the Rags modulate mTORC1 activity. These studies revealed that RagC and D can directly interact with a subset of mTORC1 substrates, TFEB and the MiT-TFE factors, which are key positive regulators of lysosomal biogenesis and autophagy, to promote their recruitment to mTORC1^28,31^. This substrate recruitment mechanism mediated by the Rags forms the basis of a previously unrecognized mTORC1 substrate specificity. Thus, the Rags control cell metabolism by modulating the switch between mTORC1-mediated anabolism and TFEB-mediated catabolism.

Considering the importance of the Rags in the regulation of cell metabolism, the observation that dysfunction of the Rags results in human diseases, herein defined as ragopathies, is not surprising. Both gain-of-function and loss-of-function mutations occurring in either the Rags or in regulators of the Rags, can give rise to ragopathies. Interestingly, while all disease-causing mutations detected in the Rags appear to be gain-of-function, the opposite is true for mutations in regulators of the Rags. However, it is important to bear in mind that some of the loss-of-function mutations occur in negative regulators of the Rags, ultimately resulting in gain-of-function of the Rags. A variety of phenotypes, affecting several organs and tissues, such as brain, heart, eye, and kidney are observed in diseases caused by Rags dysfunction, reflecting the essential role of the Rags in the metabolism of all cell types. The finding that mutations of Rags cause cardiomyopathies is consistent with the very high expression levels of the Rags in the heart. However, for other tissues such correlation between Rag expression levels and phenotype is less clear.

Dysfunction of the Rags can also promote tumorigenesis, as seen in the case of RagC mutations in follicular lymphoma and mutations of FLCN, a RagC/D activator, in BHD syndrome, which is associated with renal cancer. In both of these conditions somatic mutations play a crucial role in tumorigenesis. Interestingly, recent studies showed that in BHD syndrome TFEB and TFE3 are the main drivers of kidney cystogenesis and tumorigenesis, as well as of the mTORC1 hyperactivation associated with this condition^28^. A similar mechanism appears to operate in another inherited cancer syndrome, Tuberous Sclerosis, suggesting that the loss of function of TSC may impair Rags activity through a still unidentified mechanism^107–109^. These observations highlight the importance of determining the relevance of mTORC1 and MiT-TFE factors dysregulation in ragopathies. Consequently, when mTORC1 activity is measured in these conditions it is important to analyze not only the phosphorylation of canonical substrates such as S6K and 4E-BP1 but also the phosphorylation and subcellular localization of TFEB and TFE3.

Current treatments of ragopathies are primarily focused on symptom management and supportive care. However, the significant advancement of our understanding of both the regulation of the Rags and of Rags’ downstream effectors offers the opportunity to identify novel therapeutic targets for ragopathies. Depending on disease type, both the inhibition and activation of Rag-mediated pathways need to be considered. It is important to consider that therapeutic strategies directed towards the modulation of the Rag machinery need to be highly selective and reversible, given the relevance of this machinery in the regulation of cell metabolism. As our understanding of molecular mechanisms advances, new therapeutic strategies may emerge. This underscores the critical need for future investigations in this fascinating field.
